# Towards a neurocognitive profile in familial cerebral cavernous malformations

**DOI:** 10.1007/s13760-023-02314-x

**Published:** 2023-07-01

**Authors:** Cristiana Silva, João Durães, Marisa Lima, Daniela Jardim Pereira, Isabel Santana, Maria Rosário Almeida

**Affiliations:** 1 Neurology Department, Centro Hospitalar e Universitário de Coimbra, Praceta Professor Mota Pinto, 3004-561 Coimbra, Portugal; 2 Faculty of Medicine, University of Coimbra, Coimbra, Portugal; 3 Center for Innovative Biomedicine and Biotechnology, University of Coimbra, Coimbra, Portugal; 4 Center for Research in Neuropsychology and Cognitive Behavioral Intervention (CINEICC), Faculty of Psychology and Educational Sciences, University of Coimbra, Coimbra, Portugal; 5 Neurorradiology Functional Area, Imaging Department, Centro Hospitalar e Universitário de Coimbra, Coimbra, Portugal

**Keywords:** Genetic diseases, Neurocognitive disorders, Familial cerebral cavernous malformation, Developmental venous anomalies

## Abstract

**Background:**

Familial cerebral cavernous malformations (FCCM) is a rare autosomal dominant disease, characterized by vascular malformations that can lead to macro and microhemorrhages. The neurocognitive impact of FCCM is still underrecognized.

**Methods:**

We report the clinical, neurocognitive, imaging and genetic data of a three generation family with FCCM.

**Results:**

A 63-year-old man (proband) had progressive memory impairment since the last year. Neurologic exam was unremarkable. Brain MRI showed multiple large cavernomas (mainly in the pons, left temporal, and right temporo-parietal) and scattered microhemorrhages. Neuropsychological assessment mainly revealed left frontal and right temporo-parietal dysfunction. A 41-year-old daughter, presented with headache, vertigo and memory complaints in the last 2 years. Neurological examination revealed left central facial paralysis. Brain MRI showed two small right parietal and internal capsule cavernomas, as well as microhemorrhages. Neuropsychological assessment showed moderate temporal neocortical left dysfunction. A 34-year-old daughter had recurrent headache and memory complaints, with unremarkable neurological exam. Brain MRI revealed two large cavernomas (left fronto-orbitary and inferior temporal), with few microhemorrhages. Neuropsychological assessment was normal. A granddaughter had mild headaches and a small right cerebellar cavernoma, without microhemorrhages. Neuropsychological assessment showed mild temporal neocortical left dysfunction. A nonsense variant, c.55C > T; p.R19* generating a premature stop codon in *CCM2* gene shared by all affected family members was identified.

**Conclusions:**

Neuropsychological evaluation showed that memory complaints and cognitive impairment could be an important unrecognized finding in FCCM. Its pathophysiological mechanisms are still unknown but the role of recurrent microhemorrhages could provide an interesting hypothesis.

## Introduction

Cerebral cavernous malformations (CCM) are collections of structurally abnormal slow-flow capillaries predominantly in the central nervous system [1]. Despite rare, they account for 5–15% of all vascular malformations [1, 2]. Familial CCM (FCCM) is usually associated with multiple lesions and inherited in an autosomal dominant pattern, due to mutations in three known genes *CCM1* (*KRIT1*), which is the most common, *CCM2* (*MGC4607*), and *CCM3* (*PDCD10*) [1, 2].

About 20–50% of FCCM patients remain asymptomatic, often diagnosed after incidental findings in brain imaging [1]. The most common symptoms are seizures, intracranial hemorrhage, focal neurological deficits and headache [3]. Most lesions are supratentorial, with an annual risk of intracranial hemorrhage of 0.6–11% per patient-year [2]. Other important finding are recurrent microhemorrhages, leading to hemosiderin deposition and perilesional gliosis, believed to be a cause of seizures and being proposed as a contributing factor to neurodegeneration [1].

Despite recent advances in genetics, imaging, and biomarkers, the neurocognitive impact of FCCM is almost unreported in the literature.

## Methods

We report the clinical, neurocognitive, imaging, and genetic data in a three generation Portuguese family with FCCM due to a *CCM2* mutation. The study conforms to the Declaration of Helsinki. All subjects gave informed consent for participation in this study.

### Genetic study

The proband was screened for an NGS-customized gene panel which included the three *CCM* genes. Identified variants were evaluated for coverage and visually inspected using the Integrative Genomics Viewer. Variant annotation was performed using a multistep process workflow to individually assess variants pathogenicity, based on the American College Medical Genetics and Genomics and the Association for Molecular Pathology (ACMG/AMP) guidelines [4]. Afterwards, the pathogenic variant identified in the proband was searched for in the other family members by Sanger sequencing, to confirm the co-segregation of the variant with the disease in the family.

### Neuropsychological assessment

All patients performed a comprehensive assessment including: Montreal Cognitive Assessment (MoCA) [5], the Battery of Lisbon for the Assessment of Dementia [6] and the Trail Making Test (A and B) [7]. This battery assesses the following cognitive abilities: attention; verbal initiative, motor and graphomotor initiatives; verbal comprehension; verbal and non-verbal reasoning; orientation; visuo-constructional abilities; basic written and mental calculus; immediate memory; semantic memory, episodic visual memory; working memory; learning and episodic verbal memory. Individual test scores were converted into z-scores. Presence of impairment was considered when *z*-score < –1.

### Neuroimaging study

Brain imaging was performed in all four patients at different timepoints and in distinct MR scans (1.5 and 3 T), always including a T2 gradient-echo or, when available, a Susceptibility-Weighted Imaging (SWI).

## Results

### Clinical characteristics

The proband (II-5), a 63-year-old man, right-handed, 4 years of schooling, with type 2 diabetes and hypertension, presented with subjective memory complaints, depressed humor, and apathy in the prior year, and also nonspecific headache for 5 years. Neurological examination and initial laboratory study were unremarkable. Initial CT scan revealed multiple hyperdense brain lesions, compatible with cavernomas.

The proband’s 41-year-old daughter (III-3), right-handed, 12 years of schooling, without relevant past medical history, presented with headache and vertigo since the age of 31. Neurological examination revealed left central facial palsy and upper limb weakness (grade 4/5). Currently, she has mild headaches and memory complaints, without daily living impact.

The proband’s 34-year-old daughter (III-5), right-handed, 12 years of schooling, had a fall with head trauma at the age of 27. Subsequent CT scan revealed a left frontal lobe cavernoma and she was asymptomatic for a period of 6 years. At age of 33 she had a progressive headache and a seizure with CT scan revealing a new cavernoma in the left temporal lobe. Currently, she has mild headaches and mild memory complaints. Past medical history and neurological examination were unremarkable.

The proband’s 20-year-old granddaughter (IV-2), also presented with sporadic headache, without seizures or neurological deficits. She is currently undergoing a higher education degree without subjective memory complaints. Neurological examination was unremarkable.

**Fig. 1:**
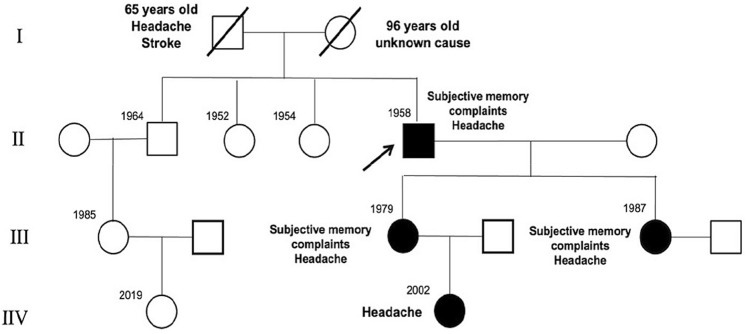
Pedigree of the family harboring *CCM2* gene mutation (variant c.55C > T p.R19*). The arrow indicates the index patient (proband), and all affected members are indicated by filled symbols. Squares represent males, and circles represent females. A diagonal line through the symbol represents a deceased individual

The pedigree chart is presented in Fig. 1.

### Genetic study

**Fig. 2:**
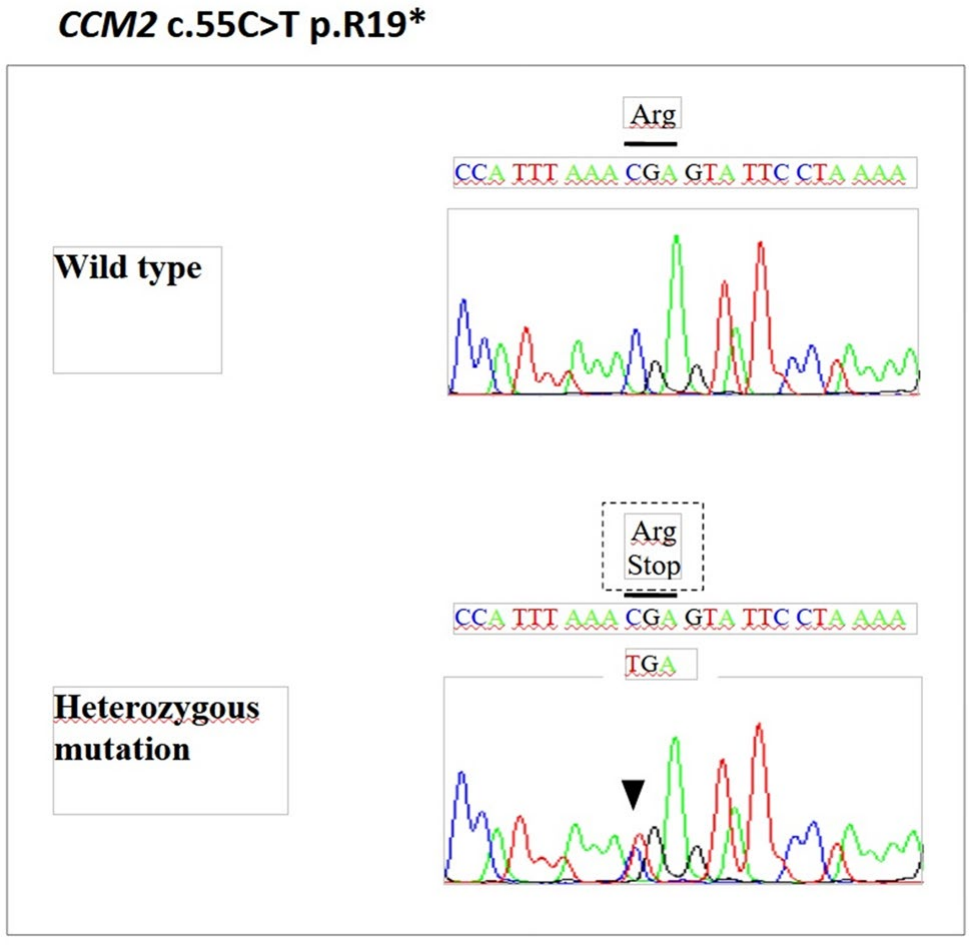
Electropherograms showing a heterozygous c.55C > T nonsense mutation in the exon 2 of *CCM2* gene with a limited reading frame depicting the corresponding amino acid substitution p.R19*, with an early stop codon gained

The genetic analysis of this family revealed a heterozygous nonsense variant in the *CCM2* gene, c.55C > T (p.R19*) (Fig. 2), previously reported in the literature in patients with FCCM [8]. Disease databases (HGMD, ClinVar) and analysis tools for prediction of variants pathogenicity (VarSome, UMD-Predictor), also support a pathogenic role of this variant in the disease phenotype. In this line, the allele frequency data (gnomAD = 0.000004, ExAC = 0.000008), also supported a damaging effect of this variant (Table 1). This variant is in a N-terminal domain, in exon 2 of the gene and causes a premature stop codon at nucleotides 55–57, leading to premature termination of the CCM2 protein at arginine 19 (p.Arg19*). Its pathogenicity was assessed according to the ACMG/AMP criteria, and was categorize as pathogenic, with the criteria of PVS1, PS4, PM2, PP1, PP4 and PP5.

**Table 1:** Pathogenic variant identified in *CCM2* gene by NGS-customized gene panel sequencing

Gene	cDNA	Predicted protein	Location	MAF		dbSNP/HGMD	Reference
				ExAC	gnomAD		
***CCM2*** NM_031443.3	c.55C > T	p.Arg19Ter	Exon 2	0.000008	0.000004	rs755800734@CM056304	[8]

### Imagiological findings

**Fig. 3:**
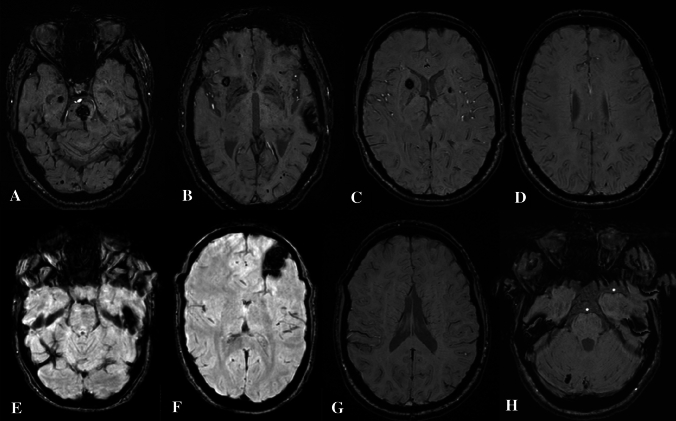
Susceptibility-weighted imaging, axial planes of the four members of the family. **A**, **B** Proband (II-2), multiple cavernomas, supra and infratentorial, the largest ones in pons (**A**) and left superior temporal lobe (**B**) and numerous type IV cavernomas. **C**, **D** Older daughter (III-3), right caudate nucleus/anterior limb of internal capsule and left lenticular nucleus (**C**), infracentimetric cavernomas located in right supramarginal gyrus (**D**), and additional type IV cavernomas mainly in the frontal region. **E**, **F** Younger daughter (III-5), two major cavernomas on left temporal superior gyrus (**E**) and fronto-orbitary region (**F**) and a pericentrimetric cavernoma with acute hemorrhage and size increasing in the left superior lobe parietal lobe. **G**, **H** Granddaughter (IV-2), one cavernoma on right cerebellum, without acute hemorrhage. No visible type IV cavernomas

Brain MRI in the proband showed multiple cavernous malformations, supra and infratentorial. The largest ones with characteristic findings of type II cavernomas, according to Zabramski classification, located in pons, anterior right insula, left corpus callosum, left superior temporal lobe, right temporo-parietal junction and right hippocampus. In addition, SWI showed uncountable punctate lesions throughout all brain representing type IV cavernomas (Fig. 3A, B).

The MRI of the older daughter showed three infracentimetric cavernomas located in right supramarginal gyrus, right caudate nucleus/anterior limb of internal capsule and left lenticular nucleus. SWI put in evidence additional type IV cavernomas mainly in the frontal region (Fig. 3C, D).

In the brain MRI of the younger daughter two major cavernomas are visible, one at left fronto-orbitary region ant other at inferior temporal lobe. A pericentimetric cavernoma on left paramedian superior parietal lobe showed acute hemorrhage on last imaging control. A smaller cavernoma was also identified on SWI on right precentral gyrus (Fig. 3E, F).

The MRI of the granddaughter revealed a small right cerebellar lesion, corresponding to a cavernoma. SWI showed no signs of type IV cavernomas (Fig. 3G, H).

### Neuropsychological assessment

The proband presented a global psychomotor delay. Objective neuropsychological exam showed a MoCA of 17/30, positive for cognitive impairment. Comprehensive neuropsychological evaluation showed moderate to severe frontal left impairment, with an emphasis in psychomotor control, verbal initiative, and verbal abstract reasoning tasks. In addition, mild episodic visual memory and copy-drawing impairment were observed, signaling a right temporo-parietal dysfunction.

The older daughter obtained a MoCA of 24/30, positive for cognitive impairment. Comprehensive neuropsychological assessment showed a moderate temporal neocortical left dysfunction, due to episodic verbal memory impairment.

The younger daughter obtained a MoCA of 25/30, negative for cognitive impairment. Comprehensive neuropsychological evaluation was normal.

The granddaughter obtained a MoCA of 27/30, negative for cognitive impairment. Comprehensive neuropsychological assessment was compatible with mild temporal neocortical left dysfunction, due to episodic verbal memory impairment. None of the patients presented with language or behavioral changes.

## Discussion

We described a Portuguese family with FCCM with a previously described nonsense mutation at amino acid 19 (p.Arg19Ter) [8] which was segregated in the family. Since this variant gives rise to a very premature termination of translation and truncated polypeptide, it is predicted to cause loss of normal protein function through nonsense-mediated mRNA decay. Indeed, the mutated CCM2 transcript is predicted to preserve only the initial part of the CCM2 N-terminal loop and a complete loss of both the N-terminal PTB domain and the C-terminal HHD domain of CCM2. Studies have shown that the prevalence of mutations in *CCM2* gene account for 19% of the FCCM cases [9], with an estimated clinical penetrance of nearly 100% [3]. Although the global prevalence of pathogenic variants in this gene has been described [8, 9], its prevalence in Portugal is still unknown.

Extensive knowledge regarding the clinical impact is essential, particularly due to recent encouraging results in a phase 2 clinical trial with propranolol in FCCM [10].

Members of this family had a classic presentation of FCCM, with an incidental diagnosis or mild and indolent symptoms [1, 3]. Unexpectedly, since it is rarely reported, three family members presented with subjective memory complaints even at a relatively young age. Detailed analysis, with comprehensive neuropsychological assessment, revealed different cognitive profiles: left frontal and right temporo-parietal dysfunction (proband), left temporal neocortical dysfunction (older daughter and granddaughter) and no cognitive deficits (younger daughter).

The first hypothesis to explain these findings would be a correlation with the major intracranial lesions. Regarding the proband, the right temporo-parietal impairment could be associated with two major lesions, namely, the right hippocampal lesion (visual memory impairment) and temporo-parietal junction (drawing dysfunction) lesions. However, the severe frontal left impairment in the proband and the neurocognitive profile of the remaining family members is not explained by this hypothesis.

Other hypothesis could be a relationship with the number and location of microhemorrhages, since the role of small vessel disease in cognitive impairment has been extensively studied. Strategically placed lesions could lead to a specific cognitive domain impairment, whereas widespread microhemorrhages could lead to impairments mainly in executive functioning, information processing and memory [11, 12]. There are several mechanisms proposed by which microbleeds influence cognitive function, both direct or indirectly, namely: direct damage to white matter tracts and interruption of strategic cortical–subcortical circuits; structural and functional disruptions alongside the lesions, promoting an inflammatory response; and an indirect vessel effect, with subsequent micro-ischemic damage [13]. Although some reports also suggest this as a possible mechanism for cognitive impairment in multiple non-familial cavernomas [14, 15], further evidence and functional studies are still needed.

In our patients, prevalence of microhemorrhages largely increased with age and seems to be an association with cognition, since the proband had the highest prevalence of microhemorrhages and the most severe neurocognitive findings. In the other hand, the youngest daughter, had the least prevalence of microhemorrhages and, despite the severity of her macrohemorrhages and symptoms, had a normal neuropsychological assessment. However, the granddaughter already had a minor cognitive dysfunction with only a single cerebellar cavernoma and without any microhemorrhages. This suggests that other factors, such as brain network dysfunction, must be important for the degree of neurocognitive involvement in FCCM.

In conclusion, we presented a family with four affected members from three generations carrying a *CCM2* gene mutation, in which detailed neuropsychological evaluation suggested that memory complaints and cognitive impairment could be an important unrecognized finding in FCCM. Although its pathophysiological mechanisms are still unknown, the role of recurrent microhemorrhages could provide an interesting hypothesis and functional imaging studies may be a helpful tool in future studies.
